# EEG Microstates in Social and Affective Neuroscience

**DOI:** 10.1007/s10548-023-00987-4

**Published:** 2023-07-31

**Authors:** Bastian Schiller, Matthias F. J. Sperl, Tobias Kleinert, Kyle Nash, Lorena R. R. Gianotti

**Affiliations:** 1 Laboratory for Biological Psychology, Clinical Psychology, and Psychotherapy, Albert-Ludwigs-University of Freiburg, Freiburg, Germany; 2 Freiburg Brain Imaging Center, University Medical Center, Albert-Ludwigs-University of Freiburg, Freiburg, Germany; 3 Department of Clinical Psychology and Psychotherapy, University of Giessen, Giessen, Germany; 4 Center for Mind, Brain and Behavior, Universities of Marburg and Giessen (Research Campus Central Hessen), Marburg, Germany; 5 Department of Ergonomics, Leibniz Research Centre for Working Environment and Human Factors (IfADo), Dortmund, Germany; 6 Department of Psychology, University of Alberta, Edmonton, Canada; 7 Department of Social Neuroscience and Social Psychology, Institute of Psychology, University of Bern, Bern, Switzerland

**Keywords:** EEG microstates, ERP microstates, Social neuroscience, Affective neuroscience, Social interaction, Neural networks

## Abstract

Social interactions require both the rapid processing of multifaceted socio-affective signals (e.g., eye gaze, facial expressions, gestures) and their integration with evaluations, social knowledge, and expectations. Researchers interested in understanding complex social cognition and behavior face a “black box” problem: What are the underlying mental processes rapidly occurring between perception and action and why are there such vast individual differences? In this review, we promote electroencephalography (EEG) microstates as a powerful tool for both examining socio-affective states (e.g., processing whether someone is in need in a given situation) and identifying the sources of heterogeneity in socio-affective traits (e.g., general willingness to help others). EEG microstates are identified by analyzing scalp field maps (i.e., the distribution of the electrical field on the scalp) over time. This data-driven, reference-independent approach allows for identifying, timing, sequencing, and quantifying the activation of large-scale brain networks relevant to our socio-affective mind. In light of these benefits, EEG microstates should become an indispensable part of the methodological toolkit of laboratories working in the field of social and affective neuroscience.

## Introduction

### Goal of the Review

**Fig. 1:**
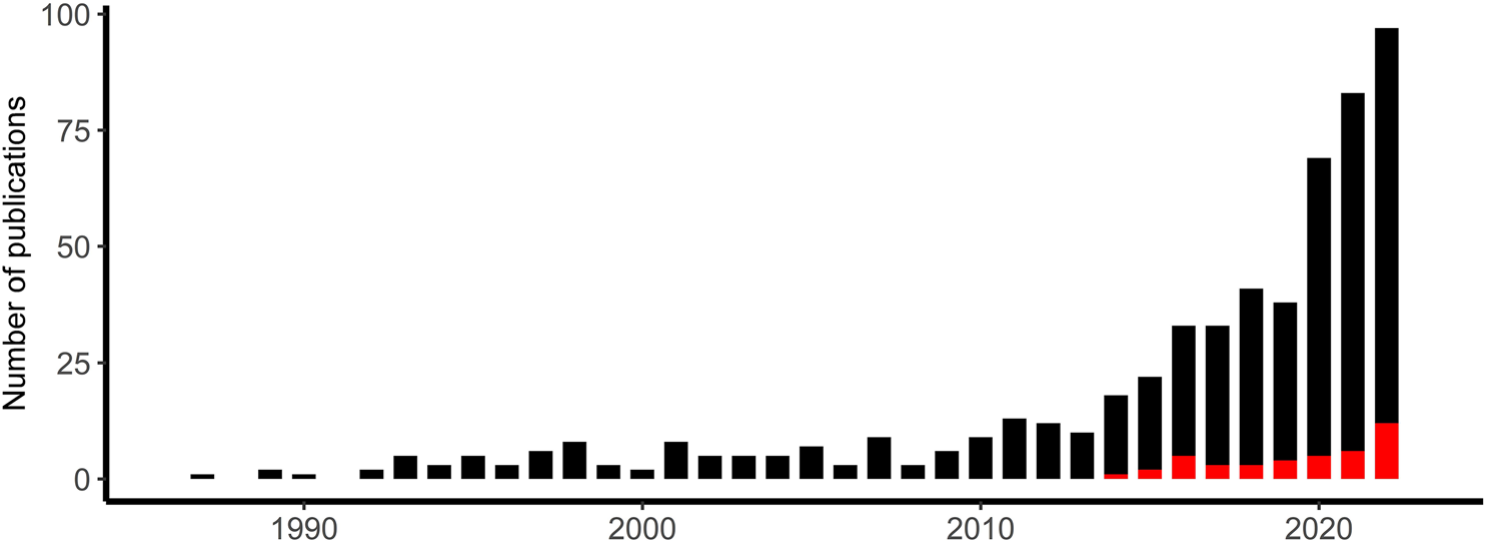
Number of microstate publications per year. The y-axis displays the number of publications as indexed in PubMed from 1987 to 2022 including the terms “EEG OR ERP AND microstates” (black bars) or the terms “(EEG OR ERP) AND (microstates AND (social OR affective))” (red bars) in the title, keywords, or abstract. Introduced by Dietrich Lehmann in 1987 (Lehmann et al. 1987), microstates initially drew limited attention within the EEG community as indexed by 126 publications over the next 25 years (1987–2012). In the last decade (2013–2022), more than  400 publications have followed. Note that only a minority (around 1/8) of all publications focused on applying microstates to ERP data

Imagine that you are in a line to board the bus to go pick up your child from kindergarten. As the bus pulls up, you notice an elderly woman down the sidewalk, burdened with shopping bags, preparing to cross the busy street. She asks you for help. You face a difficult choice. Do you help the elderly woman and miss the bus to pick up your child on time? Or do you board the bus and hope that someone else will come to her aid? This example demonstrates how social interaction requires the rapid processing of a variety of multifaceted socio-affective signals, such as eye gaze, facial expressions, gestures, physical contact, postures, and speech. Further, these signals must then be integrated with evaluations, social knowledge, and expectations stored in the brain. Researchers interested in understanding such complex social cognition and behavior are facing a “black box”: What are these distinct and rapid mental processes occurring between perceiving the bus situation described above and making the decision to help or not (e.g., conflict monitoring, neediness evaluation, planning behavior)? In addition, why might some people make the decision to help and others not? In this review, we aim to promote the analysis of electroencephalography (EEG) *microstates* (see Box 1) as an ideal approach for “opening the black box” and answering essential questions in the field of social and affective neuroscience. The microstate approach can be used to analyze both averaged EEG data (i.e., *event-related potentials*: ERPs; see Box 1) and non-averaged EEG data (i.e., *continuous EEG*; see Box 1), recorded at rest or during specific (socio-affective) states. EEG microstates provide unique information on the temporal dynamics of our socio-affective mind at a milliseconds timescale. Specifically, and in contrast to other approaches, the microstate approach allows for identifying, timing, sequencing, and quantifying the activation of large-scale brain networks that are associated with distinct socio-affective states (e.g., being stressed, because you might not be able to pick up your child on time in the example above) and that explain individual differences in socio-affective traits (e.g., general willingness to help others). The recent increase in publications indicates that more and more researchers recognize the potential of the EEG microstate approach in the fields of cognitive and clinical neuroscience (see Fig. 1; for reviews, see Galderisi and Mucci 2002; Khanna et al. 2015; Michel and Koenig 2018). However, we still see large, untapped potential for this analysis approach to better understand our socio-affective mind.

**Box 1:** Glossary

*Scalp field maps:* In topographic EEG analyses, recorded EEG data are conceptualized as a series of spatial field distributions at successive time points, which are called scalp field maps (“landscapes,” see Box 2). Changes in these topographic potential distribution maps can be interpreted as changes in the configuration of the electrical field of the brain. Topographic changes in scalp field maps over time occur step-wise and discontinuously (see *Microstates*) and are related to the activation of at least partially different neuronal populations. Note that the configuration of topographic maps is reference-independent
*EEG microstates:* Periods of time of quasi-stable EEG scalp topography, which are concatenated by abrupt transitions in the electric field configurations of the brain. Critically, electrical field properties change in a step-wise manner rather than in a continuous one. The onset and offset of microstates can be identified by segmentation procedures that have been implemented for both continuous EEG data and evoked potentials. A key strength of the microstate approach is that it does not rely on a priori selections of the reference, electrodes, or time points
*Global field power (GFP)*: The spatial standard deviation in scalp field maps indicating the overall voltage differences across all EEG channels (Skrandies 1990). GFP quantifies the amount of activity at a specific point in time. GFP does not depend on the reference chosen and can be calculated as the root of the mean of the squared potential differences in the field. GFP is typically low at the transition from one microstate to another, indicating a period during which the spatial field configuration is in a transient state
*Global Map Dissimilarity (GMD):* The stability versus changes in the spatial configuration of two electrical fields scaled to unitary strength (normalized by their GFP). High dissimilarity occurs when subsequent topographic maps change rapidly. Thus, times of high dissimilarity indicate the transition between two subsequent microstates. GMD can be calculated as the GFP of the difference map
*Event-related potentials (ERPs) vs. continuous EEG:* ERPs are short segments of brain activity time-locked to specific events of interest. Depending on the research question, typical ERP epochs comprise hundreds of milliseconds after stimulus onset. Stimulus-evoked patterns of brain activity are identified by averaging across many trials and thereby increasing the signal-to-noise ratio. In contrast, no averaging is performed when analyzing continuous EEG data. Continuous EEG data can be recorded either at rest or at specific socio-affective states of interest (e.g., while watching emotional videos)

**Box 2:** EEG waveform analysis vs. EEG brain mapping/microstates

Recorded EEG data are usually displayed as a series of waves over time in a two-dimensional matrix, with one dimension indicating data points over time (in milliseconds) and the other dimension indicating the amplitude (in microvolts) at a respective electrode (Buzsáki et al. 2012; Jackson and Bolger 2014). Typical EEG waveform analysis faces two major challenges. First, it can be difficult to justify restricting statistical analyses to only one or a few electrode sites (e.g., Murray et al. 2008). One might argue that statistical tests could be extended to a large number or even all electrode sites, but this would lead to an inflation of type 1 errors (e.g., Keil et al. 2014). Second, the selection of reference electrodes (e.g., Cz, mastoids, etc.) has a key impact on the findings of EEG waveform analysis (e.g., Yao et al. 2019). Specifically, the reference determines the level of zero voltage; thus, the voltage amplitude at all other channels will be displayed in relation to the chosen reference. Consequently, statistical analysis of EEG waveforms will be reference-dependent, making it difficult to compare findings across studies using different reference electrodes (for a visualization of this issue, see Murray et al. 2008). Even if studies rely on an identical reference electrode, any finding is dependent on the amplitude recorded at the reference, with the risk that noise at this single electrode may affect the signal in all other electrodes	
The brain mapping approach represents an alternative way to display and analyze multi-channel EEG data as a series of scalp field maps—i.e., the momentary spatial distribution of brain electrical fields (scalp topographies)—over time. Changes in scalp field maps reflect a distribution shift of active neuronal populations, mirroring changes in the activity of distinct neural networks (e.g., Vaughan 1982; Michel et al. 2004). Importantly, using a different reference electrode will, of course, change the zero line, but not the topography of the map. As a metaphor, the topography of scalp field maps is often illustrated by its “landscape,” containing characteristic “gradients, mountain peaks, and valley troughs.” The shape of a mountain range remains the same even if the height of the surface of the sea (i.e., the zero line) underneath the mountain increases or decreases (e.g., Murray et al. 2008; Michel et al. 2009). In addition, justifying the selection of individual EEG channels for statistical tests (which is often more or less arbitrary) is no longer required with the EEG brain mapping approach (note that it is also possible to use ERP microstate analysis as a data-driven technique for defining the windows of waveform analysis of well-known ERP components, for examples, see Nash et al. 2013; Schiller et al. 2023b)	

### The Basis of EEG Microstates

The EEG microstate approach originates from the “brain mapping approach,” which analyzes the series of *scalp field maps* (see Box 1) over time. Scalp field maps represent the momentary spatial distribution of brain electrical fields (i.e., scalp topographies; for a more detailed description of the brain mapping approach, see Box 2). In analyzing scalp topographies in broad-band EEG,^1^ Dietrich Lehmann and colleagues made a fundamental observation (Lehmann et al. 1987). Rather than randomly shifting over time, the scalp topographies tend to remain quasi-stable for a short period (during which the strength of the electric fields increases and decreases) and then change very quickly into a new topography, which then remains quasi-stable for another period. As different scalp topographies must have been produced by different configurations of generators in the brain (e.g., Vaughan 1982; Michel et al. 2004), it has been assumed that periods of quasi-stable topographies correspond to periods of synchronized activation of large-scale neural networks (e.g., Michel et al. 2009; Michel and Koenig 2018). Researchers reasoned that these periods may represent basic building blocks of information processing and named them “microstates”. This idea aligns with the contemporary view that brain functions involve information processing in widespread neural networks (e.g., Bressler and Menon 2010; He 2014; Fries 2015).

There are two basic steps to identify and analyze microstates, which are, despite slight methodological differences, similar for continuous EEG and ERPs (for a more detailed description, refer to Murray et al. 2008; Michel et al. 2009; Michel and Koenig 2018). First, one needs to reveal the most dominant topographies both within and across individuals by applying some sort of cluster or factor analysis (for details, see Murray et al. 2008; Michel et al. 2009). The optimal number of clusters are selected based on criteria that evaluate the quality of clustering (for an overview, see Murray 2009; Custo et al. 2017). The majority of continuous EEG studies have investigated four prototypical topographies (i.e., microstate classes) that are highly similar across studies. Though these microstate classes have been associated with activity of circumscribed neural networks, the specific function of each microstate class is still an active area of research (Michel and Koenig 2018; see Box 3 for an overview of the four prototypical microstate classes). Also note that recent research has trended to report cluster solutions with more than four classes (for a recent review, see Tarailis et al. 2023). Regarding ERPs, the number and topographies of the microstate classes depend on the task and the time window of analysis (for examples, see ERP studies listed in Table 1).

**Box 3:** The four prototypical microstate classes in resting EEG

The majority of microstate studies analyzing resting EEG have investigated four prototypical microstate classes, which typically explain 70–80% of variance in the EEG (e.g., Koenig et al. 2002). Researchers have aimed to illuminate the functional significance of these four prototypical microstate classes, as summarized below. These assumptions are based on research associating microstate classes with specific neural sources in combined EEG and fMRI studies (e.g., Britz et al. 2010) and in a source analysis approach (e.g., Custo et al. 2017^a^), and with circumscribed functions in studies using experimental manipulations (e.g., Seitzman et al. 2017). However, there is also controversial evidence regarding these assumed functions (in particular, regarding microstate class C, for details, see Tarailis et al. 2023) demonstrating the need for more research here	

Microstate class		Underlying neural sources	Assumed functions
A		Temporal regions	Auditory processing, and subject's arousal/arousability
B		Occipital regions	Visual processing
C		Anterior cingulate cortex, inferior frontal regions	Processing of self-referential internal mentation, and interoceptive-automatic processing
D		Fronto-parietal regions	Attention-related processing, and executive functioning

^a^Note that Custo et al. source-localized seven microstate classes (with the topographies of the first four classes resembling the prototypical ones).

**Table 1:** Overview of peer-reviewed studies utilizing the microstate approach in social and affective neuroscience

References	Construct of interest	Continuous EEG or ERPs	Traits or states	N^1^
Bréchet et al. (2021)	Sustained attention, meditation	Continuous	States	43
Burra et al. (2016)	Direct gaze	ERPs	States	16
Cacioppo et al. (2012)	Love	ERPs	States	20
Cacioppo et al. (2015)	Loneliness, attention	ERPs	Traits	105
Cacioppo et al. (2016)	Loneliness, attention, threat	ERPs	Traits	27
Cacioppo et al. (2018)	Lust, romantic intentions	ERPs	States	30
Chen et al. (2021)	Valence, arousal	Continuous	States	51
Decety and Cacioppo (2012)	Morality	ERPs	States	10
Du et al. (2022)	Trait anxiety	Continuous	Traits	203
Gianotti et al. (2007)	Emotional valence	ERPs	States	21
Gianotti et al. (2008)	Valence, arousal	ERPs	States	32
Globig et al. (2023)	Honesty, dishonesty	ERPs	States	150
Guo et al. (2020)	Neuroticism	Continuous	Traits	336
Han et al. (2020)	Attractiveness	ERPs	States	25
Han et al. (2022)	Attractiveness	ERPs	States	23
Hu et al. (2021)	Stress	Continuous	States	56
Hu et al. (2022)	Clustering approaches, valence	Continuous	States	32
Hu et al. (2023)	Emotional states	Continuous	States	32
Iannotti et al. (2022)	Self-other voice discrimination	ERPs	States	26
Kadier et al. (2021)	Stress	Continuous	Traits	14
Kaur et al. (2020)	Approach, withdrawal	Continuous	Traits	39
Kleinert and Nash (2022)	Aggression	Continuous	Traits	110
Kleinert et al. (2022)	Self-control	Continuous	Traits	171
Koban et al. (2012)	Cooperation, competition	ERPs	States, Traits	34
Li et al. (2021)	Disgust	Continuous	Traits	265
Liang et al. (2022)	Emotional audiovisual integration	ERPs	States	28
Liu et al. (2023)	Emotional states	Continuous	States	78
Mueller and Pizzagalli (2016)	Social threat, fear conditioning	ERPs	States	16
Nash et al. (2013)	Self-control, social decision-making	ERPs	States	45
Nash et al. (2022)	Religious belief	Continuous	Traits	69
Nash et al. (2023)	Anxiety and performance monitoring	ERPs	States, Traits	110
Ortigue et al. (2009)	Motor intentions	ERPs	States	24
Ortigue et al. (2010)	Motor intentions	ERPs	States	20
Pedroni et al. (2017)	Risk-taking	Continuous	States	39
Pegna et al. (2015)	Biological motion	ERPs	States	17
Pipinis et al. (2017)	Somatic awareness	Continuous	States	94
Pizzagalli et al. (2000)	Affective attitude, valence	ERPs	States, Traits	18
Pizzagalli et al. (2003)	Social threat, fear conditioning	ERPs	States	50
Prete et al. (2022)	Valence, emotional faces	ERPs	States	16
Schiller et al. (2023a)	Emotion recognition, social behavior, stress	ERPs	States	60
Schiller et al. (2016)	Intergroup bias	ERPs	States	84
Schiller et al. (2019b)	Oxytocin	Continuous	States, Traits	91
Schiller et al. (2020a)	Oxytocin, intergroup bias, empathy	ERPs	States, Traits	91
Schiller et al. (2020b)	Prosociality	Continuous	Traits	55
Schiller et al. (2023b)	Oxytocin, trust	ERPs	States	169
Schlegel et al. (2012)	Paranormal belief	Continuous	Traits	37
Sikka et al. (2020)	Stress	Continuous	States	50
Takehara et al. (2020)	Suppression of facial expressions	Continuous	States	25
Tarailis et al. (2021)	Somatic awareness	Continuous	Traits	202
Tanaka et al. (2021)	Subliminal affective face priming	ERPs	States	49
Thierry et al. (2006)	Human bodies, faces	ERPs	States	12
Tomescu et al. (2022)	Social imitation	Continuous	States, Traits	65
Walker et al. (2008)	Other-race face processing	ERPs	States	13
Walter and Koenig (2022)	Religious experience during worship	Continuous	States	60
Zanesco et al. (2020)	Personality, mood, attention performance	Continuous	States, Traits	227
Zanesco et al. (2021b)	Self-awareness, meditation	Continuous	States	60
Zanesco et al. (2021a)	Somatic awareness	Continuous	Traits	61
Zelenina et al. (2022)	Oxytocin	Continuous	States	20
Zerna et al. (2021)	Emotion regulation	ERPs	States	107
Zhang et al. (2021)	Empathy, disgust	Continuous	Traits	196

This table includes studies using the EEG microstate approach analyzing socio-affective states (“states”) or individual differences (“traits”) in the socio-affective mind in healthy populations

^1^Total sample size before participant exclusion

Second, the dominant topographies, which have been identified on the group-level, are then fit back to the original, individual EEG data for labeling each time point as the topography it correlates best with (in terms of *GMD*, see Box 1). Once the periods of quasi-stable topographies (i.e., microstates) are defined (usually lasting 60-120 ms for continuous EEG data: Brandeis et al. 1995, Michel and Koenig 2018; the duration of an ERP microstate is more variable and depends on the specific ERP component, ranging from very brief [< 100 ms] to several hundreds milliseconds in duration: Sur and Sinha 2009; Luck 2012), one can extract different parameters or features from each microstate class (see Box 4) to analyze associations with socio-affective traits or differences across experimental treatments or groups.

**Box 4:** Microstate parameters for statistical analysis

Microstate parameters utilized for statistical analysis	Potential interpretation regarding underlying neural network processing (Michel et al. 2009; Khanna et al. 2015; Michel and Koenig 2018)
For continuous EEG (computed for each microstate class):	
Average duration of all microstates belonging to the same class	Stability of a neural network
Frequency of occurrence (independent of its duration)	Tendency of a neural network to activate
Coverage (percentage of total time a microstate class is present)	Relative dominance of one neural network over others
Transition probabilities (of a given microstate class to any other)	Tendency of one network to activate after another network’s activation
For ERPs (computed for each microstate):	
Onset latency	Onset of neural network activation
Offset latency	Offset of neural network activation
Duration	Duration of neural network activation
Intensity (operationalized by the mean GFP)	Mean activation strength of a neural network
Area under the GFP curve	Total activation strength of a neural network

### Benefits of EEG Microstates

Compared to EEG waveform analysis, the EEG microstate approach has three key benefits. First, the microstate approach is reference-independent by analyzing temporal dynamics of scalp field maps whose topographies do not depend on the location of the reference electrode. Thereby, microstate analysis avoids a potential source of bias (i.e., the choice of the “appropriate” reference) that critically affects the findings and replicability of EEG waveform analysis. Second, the microstate approach offers unique quantifications of the EEG data with potential neurophysiological relevance that are not available in typical waveform analyses (see the parameters listed in Box 4). Third, by considering information from all available electrodes and time points, it allows a comprehensive, data-driven approach. In waveform analysis, effects may go unnoticed (e.g., during periods of low amplitude) because analyses are restricted to a few specific time windows, ERP components, and electrode positions. Moreover, as these decisions are often flexibly based on self-selected “prior research” or subjective parameters discovered via the “visual inspection of the data,” they involve a high number of researcher degrees of freedom (Murray et al. 2008; Keil et al. 2014). In comparison, the data-driven approach of EEG microstates reduces these degrees of freedom, although deciding about the cluster solution can yet affect the reported results.

## Applications of EEG Microstates

**Fig. 2:**
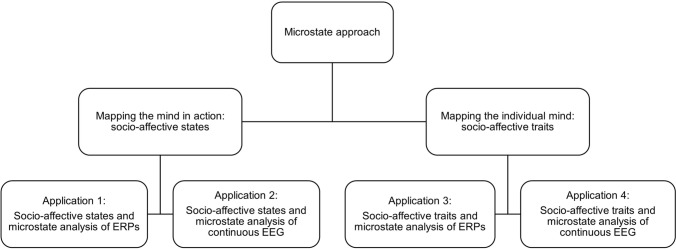
Overview of the four main applications of EEG microstates. As the first application, one can “map the mind in action” by analyzing ERP microstates evoked by particular socio-affective information. Consider the bus example described at the beginning of this review. ERP microstate analysis opens the “black box” by identifying, timing, and sequencing mental processes occurring between hearing the request by the elderly woman and making the decision to help or not (e.g., neediness evaluation, conflict monitoring, planning behavior). One can then compare these processes’ characteristics across different socio-affective states, for instance, a non-stressed state vs. a stressed state. The second application is to reveal the “socio-affective mind in action” by comparing neural network dynamics extracted from continuous EEG data across a variety of socio-affective states, for instance, during discrete emotions, or during social exclusion vs. social inclusion. The third and fourth application of EEG microstates can be subsumed under the “neural trait approach,” i.e., “mapping the individual mind” by examining socio-affective traits (e.g., Schiller et al., 2019a; for a review, see Nash et al. 2014). Briefly, this approach indexes objective information from stable brain-based characteristics to reveal the sources of individual differences in socio-affective traits (e.g., behavioral intergroup bias, emotion detection ability, prosociality, tendency to deceive others, theory of mind). Microstate parameters from both ERP data (third application) and continuous EEG data (fourth application) are promising “neural trait candidates” as they possess high retest-reliabilities (for ERP: Jouen et al. 2021; for continuous EEG: Khanna et al. 2014; Liu et al. 2020; Schiller et al. 2020b; Antonova et al. 2022; Kleinert et al. 2023) and heritability (continuous EEG: da Cruz et al. 2020)

Applying the microstate approach to ERPs and to continuous EEG offers insights into the rapid neural network dynamics underlying our socio-affective mind regarding two main research goals: i) mapping the mind in action by examining socio-affective states; ii) mapping the individual mind by examining socio-affective traits. Table 1 provides an overview of studies applying the microstate approach in social and affective neuroscience. To delineate the scope of our review, we decided to focus on studies analyzing healthy participants. Overall, there are four main applications of EEG microstates, which are illustrated in Fig. 2.

### Mapping the Mind in Action: Socio-affective States

#### Socio-affective States and Microstate Analysis of ERPs (Application 1)

**Fig. 3:**
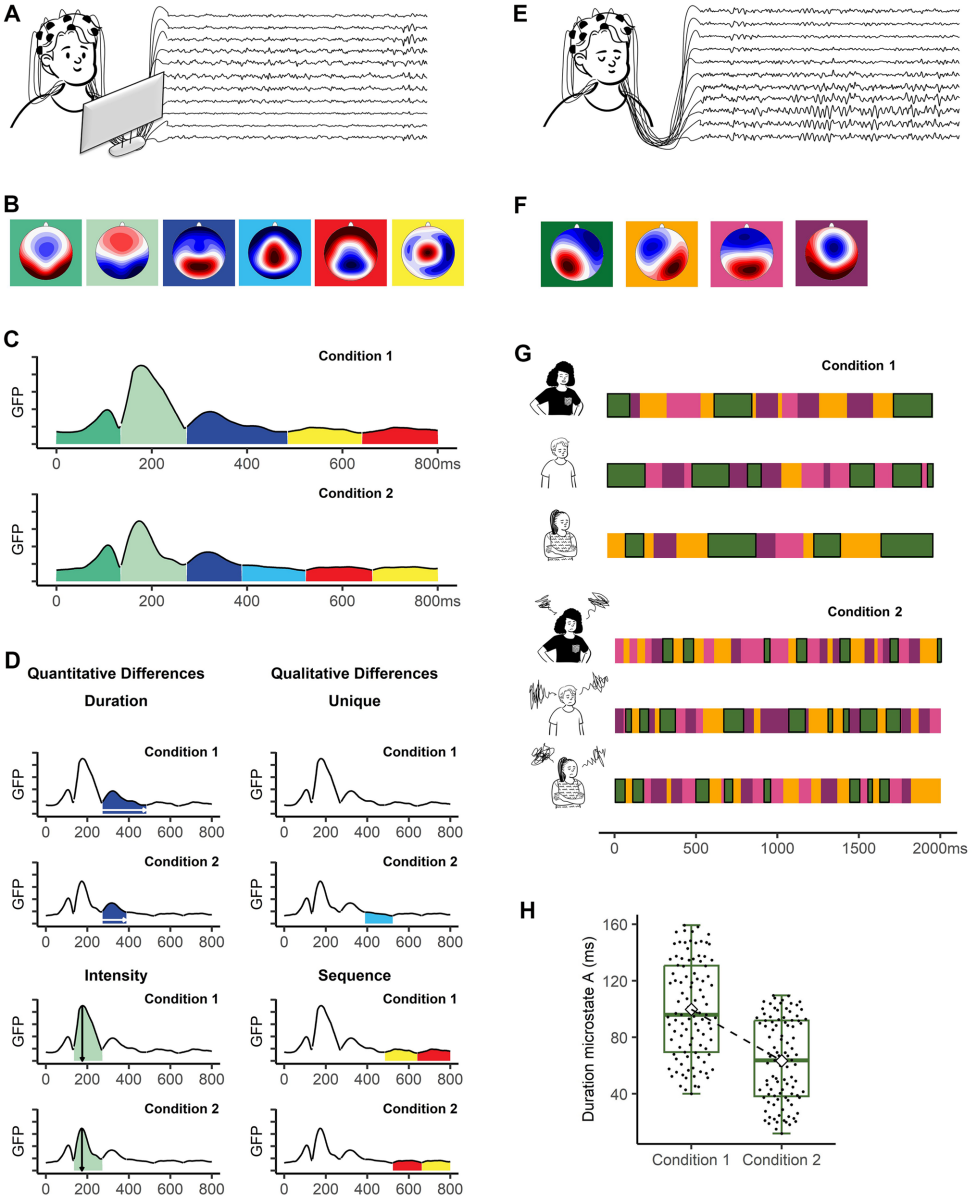
Mapping the mind in action. Examples of the application of the microstate approach in ERPs (application 1) and in continuous EEG (application 2) are shown on the left and right side, respectively. **A** ERPs are recorded while participants are confronted with specific socio-affective information (e.g., non-threatening faces vs. threatening faces, non-social vs. social stimuli). **B** Scalp topographies of 6 clusters in the sequence of their occurrence. **C** Microstates across time for two conditions plotted over Global Field Power (GFP). **D** Exemplary quantitative and qualitative differences between the two conditions. **E** Continuous EEG is recorded while participants are resting with their eyes closed. **F** Scalp topographies of the 4 prototypical microstate classes during continuous EEG: class A in green, class B in orange, class C in pink, and class D in violet. **G** Exemplary 2-s of microstates are shown for three individuals in two conditions (e.g., no stress vs. stress; happy mood vs. sad mood). All microstates belonging to class A are highlighted with a black frame. **H** Participants’ mean durations of microstate class A are shown as box plots for both conditions. The white diamond shape indicates the mean duration in the two conditions, the horizontal line the median. All figure panels are based on simulated data

Microstate analysis of ERPs enables the researcher to identify, time, and sequence neurophysiological processes across distinct experimental conditions or trial types, such as anxiety vs. no-anxiety (Nash et al. 2023), conditioned fear vs. safety stimuli (Pizzagalli et al. 2003; Mueller and Pizzagalli 2016), direct vs. averted gaze (Burra et al. 2016), honest vs. dishonest decisions (Globig et al. 2023), ingroup- vs. outgroup-related information (Walker et al. 2008; Schiller et al. 2020a), less vs. more attractive faces (Han et al. 2020, 2022), self- vs. other-voice processing (Iannotti et al. 2022), social vs. non-social stimuli/contexts (Thierry et al. 2006; Ortigue et al. 2009, 2010; Cacioppo et al. 2012, 2015, 2016, 2018; Koban et al. 2012; Decety and Cacioppo 2012; Pegna et al. 2015), stereotype-congruent vs. stereotype-incongruent information (Schiller et al. 2016), stress vs. no stress (Schiller et al. 2023a) and neutral vs. emotional stimuli (Pizzagalli et al. 2000; Gianotti et al. 2007, 2008; Cacioppo et al. 2016; Tanaka et al. 2021; Zerna et al. 2021; Liang et al. 2022; Prete et al. 2022) (Fig. 3).

Schiller et al. (2016) provide an example demonstrating *quantitative differences* across ERPs in the incongruent and the congruent condition of the Implicit Association Test (IAT), a widely-cited measure of implicit bias (Greenwald et al. 1998). This study addressed whether the IAT effect (i.e., longer response times when confronted with stereotype-incongruent information) is due to additional mental processes in the incongruent condition, or due to longer duration of the same processes. The authors identified seven microstates from stimulus presentation to response production that occurred in the same temporal sequence in both IAT conditions. However, participants showed a longer duration of one early-occurring microstate (starting around 220 ms after stimulus presentation) and of one late-occurring microstate (starting around 450 ms after stimulus presentation) in the incongruent condition, compared to the congruent condition. To shed light on the nature of the mental process underlying each microstate, the authors used source localization, linking the early microstate to lingual gyrus activity and the late microstate to activity in the middle cingulate cortex and posterior parietal areas. Based on these results, the authors suggested that the IAT effect is due to the prolongation of early-occurring perceptual processing as well as the implementation of late-occurring cognitive control, which is needed to select the correct motor response.

Response time differences in socio-affective behaviors, however, can also be due to *qualitative processing differences*. For example, Globig et al. (2023) analyzed ERPs during honest and dishonest social decision-making. Behaviorally, participants needed more time to lie than to tell the truth about the outcome of an incentivized card game. On the neural level, the authors found that the response time difference between the two conditions (lying and telling the truth) was the result of an additional microstate (occurring between 450 and 540 ms after stimulus presentation), unique to dishonest decisions, interrupting the antecedent microstate. Source localization indicated that the microstate unique to dishonest decisions was characterized by activity in the dorsolateral prefrontal cortex and the orbitofrontal cortex, whereas the antecedent microstate was characterized by activity in the supplementary motor areas. Based on the order of appearance and their pattern of neural activation, the authors speculated that when participants decide to lie, a unique process related to inhibiting the response selection process (i.e., telling the truth) occurred.

The benefit of the comprehensive ERP microstate analysis approach is also illustrated by research using classical conditioning paradigms to study brain mechanisms in the acquisition of social threat. Among the several studies on modulations of ERP components during fear conditioning, the latencies used for computing these components have varied greatly (Miskovic and Keil 2012; Ferreira de Sá et al. 2019; Sperl et al. 2021), with some focusing on early-latency (e.g., C1; Thigpen et al. 2017), others on mid-latency (e.g., N170; Camfield et al. 2016), and still others on late-latency components (e.g., LPP; Panitz et al. 2015, 2018; Bacigalupo and Luck 2018). However, as noted above, effects can easily be overlooked (e.g., during periods of low amplitude) if time windows for statistical ERP analyses are restricted to a few specific components and electrode positions (Murray et al. 2008). To overcome these methodological problems, two ERP studies applied the microstate approach to fear conditioning paradigms to help determine the electrophysiological signatures of responses to social threat (Pizzagalli et al. 2003; Mueller and Pizzagalli 2016). In these studies, the authors investigated ERPs elicited by socially-relevant conditioned stimuli (face stimuli) that had been paired with an unpleasant unconditioned stimulus (aversive noise). Critically, microstate analysis revealed that fear-conditioned faces modulated activity in visual brain regions within 80 ms after stimulus presentation (Mueller and Pizzagalli 2016). These early neural responses were overlooked in many prior studies that limited their analyses to specific (often late-latency) ERP components. This example illustrates how the comprehensive, data-driven nature of the microstate approach minimizes the risk of missing essential neurophysiological effects.

#### Socio-affective States and Microstate Analysis of Continuous EEG (Application 2)

Though relatively few in number at this point, recent work examining continuous EEG showcases the potential that microstates have for revealing the neural network dynamics of the socio-affective mind in action, at a millisecond scale, in several key ways (see Fig. 3).

*First*, microstate analysis can be combined with experimental designs (e.g., modulating the brain levels of hormones that regulate socio-affective processing: Schiller et al. 2019b; Zelenina et al. 2022; inducing psychosocial stress: Sikka et al. 2020; Kadier et al. 2021; Hu et al. 2021; performing social imitation tasks: Tomescu et al. 2022) to examine how manipulations causally change microstate parameters extracted from continuous EEG recorded after the experimental manipulation. For example, Schiller et al. (2019b) investigated the effects of experimentally increasing the availability of oxytocin in the nervous system by means of intranasal administration, compared to the administration of a placebo substance. The authors sought to illuminate the neurophysiological mechanisms underlying oxytocin’s well-known effects in the socio-affective domain (e.g., Meyer-Lindenberg et al. 2011; Ma et al. 2016). Globally, oxytocin increased stability (longer durations) across all four canonical resting networks, as recorded 45 min after substance administration (i.e., where most effective oxytocin effects are observed; see Spengler et al. 2017). Furthermore, oxytocin showed some microstate-class specific effects on further parameters, such as increasing the occurrence of microstate D (associated with attentional processing), decreasing the occurrence of microstate C (associated with interoceptive-automatic processing), and decreasing transitions from microstate B (associated with visual processing) to microstate C. The authors suggest that these results are in line with the anxiolytic effect of oxytocin to promote more attentional processing of external or social stimuli. Notably, Zelenina et al. (2022) conducted a similar study, examining the impact of oxytocin on neural network dynamics, but across a different time frame (i.e., continuous EEG was measured at various time windows, ranging from 15 to 100 min after substance administration). The results in this study partially mirror those in the Schiller et al. (2019b) study, demonstrating that, across time windows, oxytocin caused increased coverage and duration of microstates A and D, and decreased coverage of microstates B and C. The authors similarly reasoned that oxytocin seems to promote processes that tune the brain towards social stimuli.

*Second,* researchers have also manipulated socio-affective states using experimental manipulations administered across multiple sessions. For example, Zanesco and colleagues (2021) examined the impact of a 3-month meditation training on resting neural networks by means of microstate analysis (Zanesco et al. 2021b). Participants were randomly assigned to either the 3-month meditation training program or a wait-list control condition and resting EEG was recorded before and after training. Results showed that meditation increased mental awareness and quiescence, reflecting a kind of mental calm. Microstate analysis revealed six microstate classes in the continuous EEG, with four of these classes matching the prototypical microstate classes. Intriguingly, meditation training was also associated with decreased average duration across these microstates. The authors suggested that this decreased network stability reflected decreased cognitive control and increases in felt attentiveness and serenity. In a similar study, Bréchet et al. (2021) demonstrated significant topographical changes in EEG microstates in a quiet rest period after only 6 weeks of digital meditation training.

*Third*, microstate analysis can examine if different socio-affective states involve different neural network dynamics, as evidenced by unique changes to microstate features. For example, researchers have analyzed continuous EEG data recorded while participants are processing socio-affective information, such as watching emotional videos (e.g., Chen et al. 2021; Hu et al. 2022, 2023; Liu et al. 2023) or engaging in worship (Walter and Koenig 2022). For example, Hu et al. (2023) examined archival EEG data in which the original authors manipulated emotional states with the presentation of music videos. EEG was recorded during video presentation. The authors identified four microstate classes that had significant overlap with the four prototypical microstate classes. Importantly, while microstate C’s coverage and occurrence (associated with interoceptive-automatic processing) showed a positive relation with emotional arousal, microstate D (associated with attentional processing) occurred more often when watching videos of negative valence.

*Fourth*, microstate analysis can help examine mediators of subsequent behavior. For example, Pedroni et al. (2017) took the novel step of examining continuous EEG recorded during the inter-trial intervals in a risky decision-making task. On a behavioral level, results revealed that participants took higher risks after a winning trial than after a losing trial. On a neural level, microstate analysis of the inter-trial intervals revealed that two microstate classes (not referring to any of the prototypical resting EEG microstate classes) mediated the influence of outcomes of prior decisions on subsequent risk-taking on a trial-by-trial basis: one microstate class was associated with increased risk-taking, and a second microstate class was associated with decreased risk-taking. In other words, the two mediators act akin to a “gas pedal” and a “brake pedal”, respectively. Notably, the “brake” microstate class was source-localized to a bilateral prefrontal network, consistent with its role in self-regulation and cognitive control. This study demonstrates how microstate analysis can shed light on the socio-affective states that drive subsequent behavior on a single-trial level.

### Mapping the Individual Mind: Socio-affective Traits

#### Socio-affective Traits and Microstate Analysis of ERPs (Application 3)

**Fig. 4:**
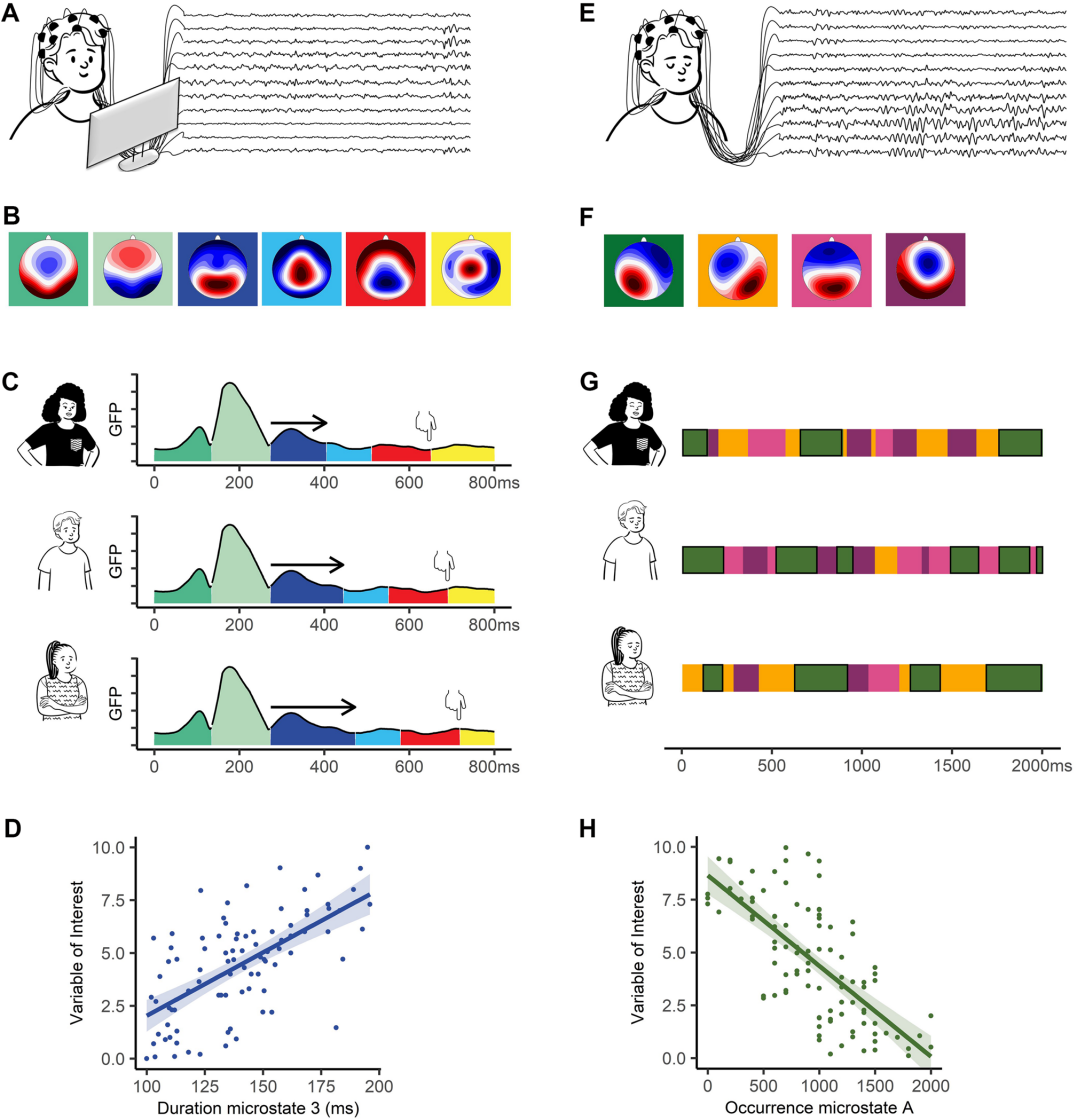
Mapping the individual mind. Examples of the application of the microstate approach in ERPs (application 3) and in continuous EEG (application 4) are shown on the left and right side, respectively. **A** ERPs are recorded while participants are viewing and responding to some specific socio-affective information (e.g., threatening faces, social stimuli). **B** Scalp topographies of the 6 clusters in the sequence of their occurrence. **C** Exemplary individual differences in microstates and response times across time plotted over Global Field Power (GFP). The hand symbols indicate mean response times. Arrows indicate the different durations of the third microstate. **D** Scatterplot of the association between duration of microstate 3 in milliseconds and the variable of interest (e.g., mean response time, indicated by the hands, in a particular condition of interest). **E** Continuous EEG is recorded while participants are resting with their eyes closed. **F** Scalp topographies of the four prototypical microstate classes during resting EEG: class A in green, class B in orange, class C in pink, and class D in violet. **G** Exemplary 2-s of microstates are shown for three individuals during resting condition. In panel **G**, all microstates belonging to class A are highlighted with a black frame. **H** Scatterplot of the association between the occurrence of microstate class A and the variable of interest (e.g., prosocial preferences, behavioral intergroup bias). All figure panels are based on simulated data

Another approach to study individual differences in socio-affective traits (e.g., behavioral intergroup bias, implicit intergroup attitudes, narcissism) is to analyze dispositional brain responses to specific socio-affective information, measured by ERPs (e.g., ingroup vs. outgroup words, Schiller et al. 2016; outcomes affecting ingroup vs. outgroup members, Schiller et al. 2020a; face attractiveness judgments, Han et al. 2022, see Fig. 4).

For example, recall Schiller et al.’s (2016) analysis of ERPs recorded during the IAT. This study revealed that response time differences in the IAT are mainly due to two microstates that take longer in the incongruent than in the congruent condition. However, what does this tell us about the mental processes underlying individual differences in implicit bias? In a next step, the authors checked whether the individual durations of these two microstates would contribute to individual differences in implicit bias. Indeed, they found that the longer an individual spent in the later occurring microstate when confronted with stereotype-incongruent information, the larger was this individual’s implicit bias. Based on the interpretation that this microstate reflected the implementation of cognitive control to select between the two competing response options, the authors suggest that individual differences in implicit bias are partly due to cognitive control ability. This finding illustrates how ERP microstate analysis can help to identify which specific features of neurophysiological processes drive heterogeneity in socio-affective traits.

Finally*,* one can also compare groups of people who already vary in socio-affective traits of interest. For example, Cacioppo et al. (2015) prescreened participants using a questionnaire on loneliness and, based on the scores, split participants into either a high or low loneliness group. These two groups then performed a Stroop task containing negative and positive words of either social or non-social relevance. Utilizing ERP microstate analysis, the authors demonstrated that individuals high in loneliness differentiate negative social stimuli from negative nonsocial stimuli more quickly than individuals low in loneliness (200 ms earlier, beginning at 280 ms after stimulus onset). Source localization analysis linked this early differentiation process to activity in brain regions associated with the orienting and executive control aspects of visual attention (e.g., extrastriate cortex, fusiform cortex, frontal eye field, dorsolateral prefrontal cortex, and anterior prefrontal cortex extending to the dorsal anterior cingulate). Relying on ERP microstates, this study demonstrated that loneliness seems to be associated with hypervigilance for social threats. This example illustrates how researchers can study individual differences in socio-affective traits by comparing millisecond brain dynamics across groups of people varying in these traits of interest.

#### Socio-affective Traits and Microstate Analysis of Continuous EEG (Application 4)

Microstate analysis of continuous EEG has been used to illuminate the sources of individual differences in socio-affective traits, in domains such as aggression (Kleinert and Nash 2022), anxiety (Schiller et al. 2019b; Du et al. 2022; Nash et al. 2023), approach vs. withdrawal tendency (Takehara et al. 2020; Kaur et al. 2020), disgust sensitivity (Li et al. 2021), empathy (Zhang et al. 2021), personality (Zanesco et al. 2020; Guo et al. 2020; Tomescu et al. 2022), prosociality (Schiller et al. 2020b), religious belief (Schlegel et al. 2012; Nash et al. 2022), or somatic awareness (Pipinis et al. 2017; Tarailis et al. 2021; Zanesco et al. 2021a). The majority of these studies have relied on regression or correlation analysis to uncover associations between features of the prototypical microstate classes (e.g., duration, occurrence, coverage, transition probabilities; see Box 4) and socio-affective traits (see Fig. 4).

For example, Schiller et al. (2020b) associated individual differences in trait prosociality with the prototypical resting-state microstates. They found that more prosocial individuals showed a higher coverage of microstate A and more transitions towards this microstate class from microstate C. The authors interpret these findings based on links of microstate A with auditory processing and microstate C with interoceptive-automatic processing (see Box 3). They suggest that more prosocial individuals might show a tendency to engage in bottom-up, sensory processing during rest, as well as a tendency to shift from stimulus-independent, top-down to more stimulus-dependent, bottom-up processing. They further suggest that these findings might be interpreted in light of the hypothesis that humans are intuitively cooperative and prosocial if they do not engage in time-consuming top-down processing during decision-making (e.g., Rand et al. 2014; but also see Kvarven et al. 2020). Overall, by identifying associations of trait prosociality with specific neural network dynamics at rest, this study illustrates how the microstate approach can help gain new insights into the sources of individual differences in socio-affective traits.

Individuals also possess more general and stable millisecond-level neural network dynamics that are independent of specific networks and that could essentially contribute to explain heterogeneity in socio-affective traits. For example, average durations and occurrences of microstates are inversely correlated across microstate classes (Khanna et al. 2014; Kleinert et al. 2022). These correlations suggest that individuals show a general tendency for more (i.e., fewer but longer-lasting network activations) or less (i.e., more but shorter-lasting network activations) network stability, potentially indicating the stability of one’s mental processing at rest. Following up on research (Kleinert et al. 2022) demonstrating a positive association between network stability and trait self-control, Kleinert and Nash (2022) found that individuals with higher levels of trait aggression (which is inversely related to self-control) showed less stable neural networks (indexed by shorter durations and more occurrences of microstates across microstate classes). In a related line of research, Tomescu et al. (2022) demonstrated more stable neural networks in individuals who are less neurotic, more conscientious, more extraverted, and report to have more coherent thoughts.

Finally, though less commonly applied, one can also illuminate heterogeneity in socio-affective traits by comparing neural network dynamics recorded at rest across two or more groups of people. This approach in particular makes sense for socio-affective traits that are more categorical in nature. For example, to illuminate why some individuals believe in deities and others do not, researchers have compared network features extracted from continuous EEG between a group of believers and a group of non-believers. They noted an increased coverage of a neural network associated with top-down processing (i.e., microstate D; Nash et al. 2022) and a decreased coverage of neural networks associated with bottom-up and interoceptive-automatic processing (i.e., microstate B, Schlegel et al. 2012; microstate C, Nash et al. 2022) in non-believers. These findings fit with the notion that believers show more intuitive reasoning and non-believers more analytic reasoning (e.g., Gervais and Norenzayan 2012). Future studies might apply this approach to investigate similarities and differences in milliseconds brain dynamics between other groups, such as people of different gender, conservatives vs. liberals, or single people vs. people in relationships.

## Outlook and Future Directions

The research summarized above illustrates how microstates can reveal the temporal dynamics of our socio-affective mind. Taking this approach could help to shed light on a range of long-standing “black box” puzzles in the field of social and affective neuroscience. For example, failed attempts to replicate popular socio-affective phenomena (e.g., ego depletion, social priming) have left large gaps within the theoretical framework of affective and social sciences (e.g., Open Science Collaboration 2015; Vohs et al. 2021). To fill these gaps, it may be necessary to study the underlying neurophysiological and mental processes during these phenomena by means of the microstate approach. For example, do self-control demands lead to prolonged mental processes in subsequent tasks, indicating more cognitive difficulty? Does social priming speed up mental processing during the presentation of target stimuli? Beyond that, we see many more fascinating research questions that could be tackled by microstate research. Do adult minds process socio-affective information more quickly compared to those of adolescents or children? Do our brains—evolutionarily optimized for interacting “face-to-face” (e.g., Kock 2004)—process socio-affective cues differently in digital environments? Are neural networks activated in the same sequence at distinct states of consciousness (e.g., distinct sleep stages; Bréchet et al. 2020; Diezig et al. 2022)? Finally, EEG microstates might not only be useful for understanding our socio-affective mind, but also for modulating it. Preliminary evidence indicates that microstate-neurofeedback training is feasible in healthy participants (Diaz Hernandez et al. 2016; Asai et al. 2022). One could thus experimentally alter microstate features to induce specific socio-affective processes, e.g., experimentally increasing a key microstate’s duration at rest to decrease trait-levels of anxiety.

## Conclusion

In sum, EEG microstate analysis offers a powerful tool for opening the “black box” of neurophysiological processing underlying our socio-affective mind. This review has illustrated how studies utilizing the microstate approach have illuminated the sources of socio-affective traits and examined distinct socio-affective states, highlighting issues of prosocial behavior, emotional processing, and social evaluations, as just a few examples. Given the major benefit afforded by EEG microstates for identifying, timing, sequencing, and quantifying the neural network dynamics underlying our socio-affective mind at a milliseconds time scale, the microstate approach has the potential to become an indispensable part of the methodological toolkit of laboratories working in the field of social and affective neuroscience.

## Footnotes

1 Recent studies have also performed microstate analysis separately for different frequency bands, see, e.g., Férat et al. (2022).
